# Unintended and Accidental Exposures, Significant Dose Events and Trigger Levels in Interventional Radiology

**DOI:** 10.1007/s00270-020-02517-2

**Published:** 2020-05-20

**Authors:** Werner Jaschke, Gabriel Bartal, Colin J. Martin, Eliseo Vano

**Affiliations:** 1 Department of Radiology, Medical University of Innsbruck, Anichstrasse 35, 6020 Innsbruck, Austria; 2 Present Address: Tel Aviv, Israel; 3 Department of Clinical Physics and Bio-Engineering, Gartnavel Royal Hospital, University of Glasgow, Glasgow, G12 0XH UK; 4 Department of Radiology, Medical Physics, Faculty of Medicine, Complutense University, 28040 Madrid, Spain

**Keywords:** European basic safety standards, Substantial radiation dose, Trigger levels

## Abstract

Over recent years, an increasing number of fluoroscopically guided interventions (FGIs) have been performed by radiologists and non-radiologists. Also, the number of complex interventional procedures has increased. In the late nineties, first reports of skin injuries appeared in the literature. The medical community responded through increased awareness for radiation protection and public authorities by recommendations and legislation, for example, the European Basic Safety Standards (EU-BSS) which were published in 2014, or the international Basic Safety Standards (BSS). Implementation of the EU-BSS requires concerted action from interventionalists, radiographers, medical physics experts and competent national authorities. Interventionalists should play an important role in this project since implementation of the EU-BSS will affect their daily practice. This paper discusses some important issues of the EU-BSS such as unintended and accidental radiation exposures of patients, the meaning of significant dose events and how to deal with patients who were exposed to a substantial radiation dose with the risk of tissue injuries. In addition, this paper provides practical advice on how to implement alert and trigger levels in daily practice of FGIs in order to increase patient safety.

## Introduction

Tissue reactions (also called deterministic effects) were first described in physicians using X-rays for medical imaging and in patients treated by X-rays for various health conditions such as naevi and tumors [1]. After initiating appropriate radiation protection measures, publications on tissue reactions in radiologists significantly reduced. Due to technical advances and the improved understanding of radiobiology, patient doses from X-ray-based imaging procedures decreased over time, until they were far less than the critical skin dose level of 3 Gy. However, starting in the early 80 s, CT and fluoroscopy became more widely used for diagnostic and interventional procedures. Over time, the interventions that could be performed under the guidance of X-ray imaging became more complex, requiring longer exposure times.

In the early 1990s, reports of skin injuries and loss of hair related to fluoroscopically guided interventions (FGIs) started to appear in the literature [2, 3]. Most radiation-induced skin injuries were reported in coronary interventions [4, 5]. These reports attracted growing attention from medical specialists, the public, regulators and radiation protection authorities. In order to improve radiation safety and contribute to the reduction of incidents in medical exposures, European Basic Safety Standards (EU-BSS) [6] and the international Basic Safety Standards (BSS) [7] were published in 2014. In the EU-BSS, the terms “accidental and unintended medical exposures” were introduced. The EU-BSS define an accident as “any unintended event, including operating errors, equipment failures and other mishaps, the consequences or potential consequences of which are not negligible from the point of view of protection or safety.” “Unintended exposure” means a medical exposure that significantly differs from the medical exposure intended for a given purpose. “Accidental medical exposure” relates to imaging of a wrong body part or imaging the wrong patient. Such exposures are referred to as “significant events” in articles 63 and 96 of the EU-BSS [6, 8]. In the case of interventional radiology, they relate to unnecessary overexposures of individual patients with the risk of tissue reactions, high effective doses or high organ doses [6, 8]. Accidental exposure may occur if the intervention is performed in the wrong region of the body, for example, the unaffected limb. Usually standards of practice require the clear identification of patients and the body region prior to any intervention, thus avoiding accidental exposure of a wrong patient or wrong part of the body [9]. For unintended exposures, no threshold dose levels are given in the EU-BSS, but a full article (art. 63) is included in the directive with the obligations of Member States of the European Union to declare these events to the competent authority, to inform the referrer, practitioner and patient, to implement corrective measures if appropriate, and to disseminate the relevant radiation protection information regarding lessons learned [6, 8].

Implementation of the EU-BSS into daily practice is in part the responsibility of interventional radiologists or any other interventional practitioner, radiographers and medical physics experts (MPEs), in addition to the hospital management and national competent authorities in Europe. Interventional radiologists should initiate steps to implement the most important rules of the EU-BSS adapted to their practice. The MPE is defined as a medical physicist having the knowledge, training and experience to act or give advice on matters relating to radiation physics applied to medical exposure, whose competence in this respect is recognized by the competent authority [6].

The goal of this paper is to provide guidelines for interventional radiologists (practitioners) on how to recognize significant dose events and how to identify patients at risk of radiation-induced tissue reactions. The most complex challenge is the management of patients unintentionally and/or unavoidably exposed to specific high dose levels. In addition, this publication provides guidelines on how to avoid significant dose events or accidental or unintended exposures of patients.

## Training of Operators

 Prior to granting the privilege to use fluoroscopy or CT, all operators should meet institutional requirements for performing FGIs or CT-guided interventions (CTGIs). All operators must receive initial training in patient radiation management when beginning work in the interventional radiology suite [10]. Radiation safety training should be in accordance with institutional policy and governmental regulations and generally will include review of the potential adverse effects of radiation on patients and staff, safe operation of the institution’s fluoroscopic or CT equipment, evaluation of factors that affect patient and staff doses, and measures that can be taken to reduce dose. Operators should be informed how to estimate patient dose using the DICOM Dose Reports or other surrogate parameters of radiation exposure [11, 12]. Close cooperation with MPEs (where available) is recommended to advise on dose levels delivered by different equipment program options. Training should also include understanding of the most important tissue reactions and the radiation dose levels, at which they may occur [3].

Operators should undergo adequate training in interventional techniques before performing interventional procedures and should be supervised by an experienced operator until they are able to perform procedures by themselves.

Medical simulators may also be used for training in catheterization techniques and how to manage radiation dose [13, 14]. One of the most reliable, scientifically validated concepts that meets the requirements for safe performance of procedures is medical simulation [13, 14]. Both trainees and experienced interventional radiologists must maintain a constant awareness of radiation dosage and opportunities to minimize exposure for patients and personnel. Simulators enable training to be undertaken repeatedly, allowing trainees to increase their procedural efficiency, and thus reduce complication rates, and ideally, radiation exposure [15, 16].

## Potentially High Dose Procedures

Radiation risks associated with interventional procedures should be discussed with patients as part of the preprocedure consent process, particularly when the expected dose of radiation may be high. Specifically, but not exclusively, the following procedures have been associated with an increased occurrence of a substantial radiation dose (Table 1).

**Table 1:** Procedures with a potentially high dose of patients

1	Transjugular intrahepatic portosystemic stent shunt (TIPSS)
2	Percutaneous biliary drainage/ stenting ± biopsy, stone removal, rendezvous maneuver
3	Hepatic chemoembolization/ abdominal arterial embolization
4	Pelvic arterial embolization
5	Thoracic and/ or abdominal EVAR
6	Neuroembolization/head (arteriovenous malformation [AVM], aneurysm, tumor)
7	Neuroembolization/spine (AVM, aneurysm, tumor)
8	Mechanical thrombectomy (stroke)
9	Selective internal radiation therapy (SIRT/Y-90 radioembolization)
10	Transcatheter aortic valve implantation (TAVI)
11	Percutaneous coronary interventions (Coronary stenting, ablation, etc.)
12	Percutaneous vertebroplasty and kyphoplasty

In addition, dose and risk may require special attention in the following cases:

- where patient weight is greater than 120 kg
- in interventions in pediatric and young adult patients involving substantial absorbed dose to radiosensitive organs (e.g., lens of eye, breasts, gonads, thyroid);
- during pregnancy
- in procedures anticipated to be technically difficult, unusually prolonged,
- in FGIs performed in the same region within the previous 3 months
- in patients with increased radiosensitivity (especially due to younger age or genetic predisposition for details, see [17–19])
- where radiation therapy has been used or is planned for the same anatomic region.

Tissues at risk are the skin, hair and during neurointerventions the lenses of the eyes [17, 20, 21]. Stochastic effects are related to effective dose, for example, to the ovaries during pelvic embolization or the thyroid during interventions in the head and neck region. However, in the vast majority of cases the effective dose is well below the critical effective dose level at or above 100 mSv [22]. Nevertheless, for some rare procedures effective dose at or above 100 mSv may be applied [23, 24]. These patients should be informed about the potential stochastic risks. How to assess and communicate stochastic risks to patients is discussed in detail elsewhere [25, 26].

## How to Measure and Report Dose

The dose parameters of modalities are always based on physical units and quantities in the header of DICOM images or in the DICOM Radiation Dose Structured Report (RDSR). The RDSR is usually part of the electronically stored images at the beginning or end of the image sequence. The most important dose quantities in FGIs are air kerma area product (also named as dose area product), *P*_KA_, and cumulative air kerma at the patient entrance reference point, *K*_a,r_ [11, 27].

Reporting criteria for significant dose events should be based on physical quantities and not on effective dose or text-based criteria like “significantly higher” [8]. State-of-the-art fluoroscopic equipment reports the before mentioned dose parameters separately for fluoroscopy and radiographic/angiographic images and for every dose event (fluoroscopy scene/angiographic series). This is important for optimization since it provides the operator with a good understanding of which exposure events (fluoroscopy/ angiographic series) and settings contributed most to radiation exposure of the patient. For estimating dose to the patient, the most important parameters are the cumulative values expressed for *P*_KA_ and *K*_a,r_ which are the sums of all dose events during the procedure. Total fluoroscopy time (FT) and total number of radiographic/angiographic images are also reported, but should not be used to identify significant dose events [8, 11].

## Trigger Levels, Substantial Radiation Doses, Alert Levels, Significant Events and Diagnostic Reference Levels

A “trigger level” or “substantial radiation dose level” (SDRL) is an appropriately selected reference value, usually of the cumulative air kerma at the patient entrance reference point (*K*_a,r_), indicating an increased risk of tissue reactions on the skin. When cumulative *K*_a,r_ for an individual patient or individual examination reaches this reference value, additional actions should be triggered. The National Council of Radiation Protection (NCRP/USA) Report 168 [28] used the term “substantial radiation dose level” (SRDL) for the reference value, and the International Commission on Radiological Protection (ICRP) used the term “trigger level” for the reference level to give a notification of potential risk [29]. The ICRP term “trigger level” will be used in the remainder of this document, and doses delivered that are above the trigger level will be referred to as substantial radiation doses.

The trigger level is set in relation to the threshold dose for tissue reactions at which 1% of all individuals exposed demonstrate the expected tissue reaction [30]. Threshold dose values recommended for patient follow-up in a consensus paper providing guidelines on patient radiation dose management and endorsed by the Cardiovascular and Interventional Radiology Society of Europe (CIRSE) and the Society of Interventional Radiology (SIR) are given in Table 2 as trigger values.

**Table 2:** Trigger levels^a^ according to CIRSE Guidelines [11]

Dose metric	Trigger value^b^
Peak skin dose (*D*_skin, max_ or PSD)	3 Gy
Cumulative air kerma at a reference point (*K*_a,r_)	5 Gy
Air kerma area product (*P*_KA_) (assuming a 100 cm^2^ field at the reference point)	500 Gy cm^2^
Fluoroscopy time (only if PSD, *K*_a,r_ and *P*_KA_ are not available)^c^	60 min

^a^The radiation dose level that is intended to trigger follow-up for an FGI procedure, in order to ensure detection of any clinically relevant injury in an average patient

^b^These criteria apply to radiation dose values at the end of a procedure

^c^Facilities performing potentially high-dose FGI procedures shall measure dose metrics and should not rely on fluoroscopy time alone

These are similar to the SRDL values in NCRP report 168 [28], and since they may cause a clinically relevant injury in an average patient, they should initiate follow-up and monitoring of the patient. However, since there are substantial differences in radiosensitivity, the expected tissue reactions will only occur at higher dose levels in most individuals.

Alert levels may be set below the trigger level in order to notify the interventionalist that he/she is approaching the trigger level [31] so that further optimization of the procedure can be considered at an earlier stage [31]. On such an occasion, it may be appropriate to consult a more experienced interventionalist. If an alert or trigger level is exceeded while performing an FGI procedure, the interventionalist can decide to modify the radiation exposure or interventional procedure to avoid an excessive dose to the patient or to continue with the procedure if the benefit for the patient outweighs the risk. If patient dose is at or above a trigger level, then the interventionalist shall place a note of the exposure in the medical record after completing the procedure, and the patient should be followed up [11].

Recently, the International Atomic Energy Agency (IAEA) introduced an anonymous self-reporting system (SAFRAD = SAFety in RADiological procedures reporting system), which uses the same trigger values [32]. SAFRAD asks radiology and cardiology departments to report cases in which a trigger event is encountered (Table 3).

**Table 3:** SAFRAD reporting criteria [32] 1 and 5 should only be used if 2 and 3 are not available!

*Trigger values*	
Fluoroscopy time	> 60 min
Air kerma area product (or dose area product)	For cardiac and neurological procedures: > 300 Gy.cm^2^ or 30,000 cGy.cm^2^ or 30,000 µGy.m^2^ or 300,000 mGy.cm^2^ For other procedures: > 500 Gy.cm^2^ or 50,000 cGy.cm^2^ or 50,000 µGy.m^2^ or 500,000 mGy.cm^2^
Cumulative air kerma (dose) at the interventional reference point	> 5 Gy or 5000 mGy
Measure peak skin dose	> 3 Gy or > 3000 mGy
Number of cine acquisition series	> 20
*Trigger events*	
Observed radiation injury	
Patient had multiple procedures within the last 12 months	
Wrong procedure performed	
Procedure performed on the wrong patient	
Unknown pregnancy at time of procedure	

The purpose of a follow-up clinical examination (usually 2–4 weeks and 6 months after the procedure) is to detect skin effects that may require further management or prolonged follow-up (Table 4). Indicative values of peak skin dose related to the threshold for skin effects, together with ranges for measurable dose quantities at which these might occur, are included in Table 4 as a guide for operators. In case of a minor skin reaction, such as transient erythema, the patient should be asked to evaluate the skin reaction in the region of the erythema and, if necessary, document (for example, by photography) and report skin changes to the operator/caring physician. It is important to note that skin injuries at 5 Gy *K*_a,r_ are rare in body and cardiac interventions. *K*_a,r_ is not identical with peak skin dose (PSD) in body and cardiac interventions since the position of the radiated field of view changes during the procedure and the interventional reference point is not identical with the level of the skin in most positions of the C-arm. Maccia et al. [33] reported in 2015 no long-term (> 1 year) skin injury despite a follow-up of patients when *K*_a,r_ was > 7,000 mGy . However, Guesnier-Dopagne et al. [34] reported a much higher incidence of acute (approx. 9%) and chronic radiodermatitis (approx. 20%) in patients exposed above trigger levels if a systematic follow-up was implemented and performed by dermatologists. This may indicate that a lot of radiation-induced skin changes are not detected by routine follow-up. In addition, some patients developed chronic radiodermatitis after more than 2 years without a previous acute radiodermatitis [34]. A recent publication reported that hair thinning and hair loss occurred in 15–25% of patients receiving a cumulative *K*_a,r_ of 3.5–4 Gy during neurointerventions [35]. Thus, in neurointerventions cumulative *K*_a,r_ may be closer to peak skin dose, because of overlapping fields. The trigger level in neurointerventions should, therefore, be 3 Gy cumulative *K*_a,r_.

**Table 4:** Threshold values of measurable dose quantities for which there are risks of skin effects following IR procedures

Peak skin dose (Gy)	*K*_a,r_(Gy); body	*P*_KA_ (Gy cm^2^); body	*K*_a,r_(Gy); head	Risk of tissue reaction
2–3	3–8	150–300	2–5	Tissue reactions unlikely to occur
3–5	5–12	300–800	3–8	Small risk of transient erythema and epilation. Recovery from hair loss
5–8	8–20	400–1200	5–12	Risk of erythema and epilation in some patients. Effects may appear within 2–8 weeks Erythema may be prolonged
8–12	12–30	600–2000	8–16	Transient erythema expected as a prompt effect. Skin desquamation, prolonged epilation

Note: threshold values are different for cumulative air kerma (*K*_a,r_) and air kerma area product (*P*_KA_) for FGIs in the body and head, but peak skin dose levels are identical!

The EU-BSS [6] requests that a reporting system is set up by every member state. However, in most EU member states protocols have not been implemented giving information about how to report and which events to report. Until national regulations and codes of practice derived from transposition of the EU-BSS are implemented, we recommend significant events at or above the trigger levels are documented locally and necessary steps are initiated as mentioned below. Reporting of significant events to the SAFRAD database is also recommended (see above) [32].

According to Martin et al. [31], the following steps should be initiated to investigate causes and to implement measures to prevent further overexposures:

- Calculate or estimate the doses received and the dose distribution within the patient.
- Indicate the corrective actions required to prevent recurrence.
- Implement all the corrective actions that are under the registrant’s responsibility
- Produce and keep a written record that states the cause of the incident, in addition to records required by the regulatory body.
- Ensure that the appropriate radiological medical practitioner informs the referring medical practitioner and the patient or the patient’s legal authorized representative of the unintended or accidental medical exposure.

There have been suggestions about the use of diagnostic reference levels (DRLs) in these procedures, but it should be noted that these are not intended to be used for individual patients or procedures [29]. National DRLs are derived from distributions of median values of measured dose quantities from patient dose surveys performed in individual hospitals throughout a country in order to provide indicative values of patient dose levels. Local typical dose values from surveys of groups of patients should be compared with national or local DRLs on a yearly basis. If no national DRLs for a given procedure are implemented, local DRLs should be established. DRLs are usually updated on a regular basis (for example, at intervals of 3–5 years) by national authorities. Thus, DRLs provide a useful indication of expected dose levels for standard diagnostic and interventional procedures.

### Peak Skin Dose

Peak skin dose (PSD) cannot be easily measured or calculated. Currently, it is still not routinely reported on angiographic equipment. Some vendors provide skin dose maps or provide software which calculates maximum skin dose to the back of the patient [36, 37]. In the future, PSD will probably be part of the RDSR. The trigger level for the skin, above which additional calculations should be considered with the involvement of a MPE, is usually 3 Gy [8]. For doses below this level, surrogate parameters such as *P*_KA_ or *K*_a,r_ should be sufficient for estimating skin dose.

### Eye Lens Dose

The threshold for radiation injuries to the eye lens is about 500 mGy [30]. As for the skin, eye lens dose cannot be calculated or measured easily. A critical eye lens dose may be reached in complex neurointerventions with biplane angiographic equipment. The critical organ is the lens of the left eye of the patient. In these interventions, dosimeters positioned close to the left eye may be used to estimate the eye lens dose [20]. *P*_KA_ of the lateral plane can be used for risk assessment. According to Sanchez et al., the trigger level suggesting follow-up for potential lens injuries in the left eye is *P*_KA_ > 300 Gy cm^2^ (lateral plane) in neurointerventions [20]. In this study, there was a likelihood of 47% that the lens dose of the left eye was at or above the threshold dose of 500 mGy.

## Dose Management Systems

The DICOM dose report or other equivalent documents shall be used to evaluate the doses to patients at the end of each procedure. However, since patients may have been exposed to radiation in the same region of the body previously, a cumulative dose should be readily available for the interventionalist prior to the procedure. Dose management systems can provide this information. Thus, exposures above a defined trigger level can be easily identified, and in case of prior exposures, the next procedure can be planned trying to avoid further exposure of the area irradiated previously. In addition, dose management systems help to optimize the interventional procedures using the technical, geometrical and dosimetric information contained in the radiation dose structured reports. If these systems are still not available, it is suggested to follow the recommendations of the SIR and CIRSE “Guidelines for Patient Radiation Dose Management” [11].

## Summary

The risk of accidental and unintended exposures in IR is extremely low, but if they occur, the interventionalist has to document the event and the dose erroneously applied to the wrong part of the body or wrong individual or any repetition of radiation exposures due to technical errors or patient/side confusion. These exposures may occur due to technical problems (for example, wrong settings of angiographic equipment), due to operator negligence, due to unexpected difficulties/complications during the procedure or due to medical error (exposing wrong patient/ body region). This has to be documented and an explanation included of why the unintended exposure occurred and how this will be avoided in the future.

In addition, special attention has to be paid to patients who have received a significant skin dose and are likely to have to undergo additional procedures in the same region of the body. This may result from the complexity of the procedure rather than any mistake or accident. Cumulative dose from all such procedures should be documented in the patient record, radiology report and readily accessible prior to further procedures being performed.

In the case of interventional radiology, the term “significant events” is related to radiation exposures of individual patients with a relevant risk of tissue reactions, high effective dose or high organ doses. The interventionalist has to define trigger levels according to international/national recommendations for notification of high doses. An alert level may be set at a lower dose to warn the interventionalist of the potential high dose at an earlier stage. A radiation exposure above a trigger level requires specific justification (for example, life saving, complex procedure), documentation and eventually follow-up of the patient. In addition, the interventionalist should analyze the event together with a MPE to suggest optimization strategies.

Prior to the procedure, informed consent has to be obtained from the patient. The risks from the procedure and the radiation exposure should be explained and discussed with the patient. After the procedure, the patient, a family member or other caregivers have to be informed if a substantial radiation dose at or above the trigger level was encountered and what risks are associated with the applied dose. It is recommended that doses are given in terms of cumulative air kerma (*K*_a,r_) and air kerma area product (*P*_KA_) and the potential associated risks are stated in the radiology report. In case of pediatric interventions, the higher risk of stochastic effects has to be considered.

DRLs can provide an indication of expected dose levels for radiology procedures, but still do not exist for many interventional procedures. However, local typical dose values (median values) should be compared with national or local DRLs annually and if no national DRLs are defined for a given procedure, local DRLs could be established.

Radiation protection is a continuous career long process for interventional radiology staff, not an occasional event. Clearly, profound understanding of radiation protection in image guided interventions has a potential to reduce an irrational fear, thus attracting medical students and young physicians to the exciting field of IR.

## Abbreviations

CIRSE: Cardiovascular and Interventional Radiology Society of Europe
CT: Computed tomography
CTGI: CT guided intervention
DICOM: Digital Imaging and Communications in Medicine
EU-BSS: European Basic Safety Standards
EFOMP: European Federation of Organisations for Medical Physics
EFRS: European Federation of Radiographers Societies
ESR: European Society of Radiology
EU: European Union
EURATOM: European Atomic Energy Community [EAEC])
FGI: Fluoroscopically Guided Intervention
IAEA: International Atomic Energy Agency
ICRP: International Commission on Radiological Protection
IR: Interventional radiology
MPE: Medical physics expert
NCI: National Cancer Institute
NCRP: National Council on Radiation Protection and Measurements
PSD: Peak skin dose
RDSR: Radiation Dose Structured Report
SAFRAD: SAFety in RADiological procedures reporting system
SIR: Society of Interventional Radiology
SRDL: Substantial radiation dose level
